# Symptomatic and Disease-Modifying Therapy Pipeline for Alzheimer’s Disease: Towards a Personalized Polypharmacology Patient-Centered Approach

**DOI:** 10.3390/ijms23169305

**Published:** 2022-08-18

**Authors:** Xavier Morató, Vanesa Pytel, Sara Jofresa, Agustín Ruiz, Mercè Boada

**Affiliations:** 1 Research Center and Memory Clinic, Fundació ACE, Institut Català de Neurociències Aplicades, Universitat Internacional de Catalunya, 08017 Barcelona, Spain; 2 Centro de Investigación Biomédica en Red de Enfermedades Neurodegenerativas (CIBERNED), Instituto de Salud Carlos III, 28029 Madrid, Spain

**Keywords:** Alzheimer’s disease, Aβ, tau, disease-modifying therapies, inflammation

## Abstract

Since 1906, when Dr. Alois Alzheimer first described in a patient “a peculiar severe disease process of the cerebral cortex”, people suffering from this pathology have been waiting for a breakthrough therapy. Alzheimer’s disease (AD) is an irreversible, progressive neurodegenerative brain disorder and the most common form of dementia in the elderly with a long presymptomatic phase. Worldwide, approximately 50 million people are living with dementia, with AD comprising 60–70% of cases. Pathologically, AD is characterized by the deposition of amyloid β-peptide (Aβ) in the neuropil (neuritic plaques) and blood vessels (amyloid angiopathy), and by the accumulation of hyperphosphorylated tau in neurons (neurofibrillary tangles) in the brain, with associated loss of synapses and neurons, together with glial activation, and neuroinflammation, resulting in cognitive deficits and eventually dementia. The current competitive landscape in AD consists of symptomatic treatments, of which there are currently six approved medications: three AChEIs (donepezil, rivastigmine, and galantamine), one NMDA-R antagonist (memantine), one combination therapy (memantine/donepezil), and GV-971 (sodium oligomannate, a mixture of oligosaccharides derived from algae) only approved in China. Improvements to the approved therapies, such as easier routes of administration and reduced dosing frequencies, along with the developments of new strategies and combined treatments are expected to occur within the next decade and will positively impact the way the disease is managed. Recently, Aducanumab, the first disease-modifying therapy (DMT) has been approved for AD, and several DMTs are in advanced stages of clinical development or regulatory review. Small molecules, mAbs, or multimodal strategies showing promise in animal studies have not confirmed that promise in the clinic (where small to moderate changes in clinical efficacy have been observed), and therefore, there is a significant unmet need for a better understanding of the AD pathogenesis and the exploration of alternative etiologies and therapeutic effective disease-modifying therapies strategies for AD. Therefore, a critical review of the disease-modifying therapy pipeline for Alzheimer’s disease is needed.

## 1. Introduction

Alzheimer’s disease (AD) is the most common form of dementia in the elderly, affecting approximately 50 million people worldwide [1]. This number is expected to double in the next 20 years, reaching 150 million by the middle of the century [2]. Importantly, the concept of AD has changed to recognize that AD is a continuum with a long preclinical phase of Subjective Cognitive Decline (SCD), a stage of mild cognitive impairment (MCI), and a dementia phase [3,4]. AD is characterized by memory and neuronal loss, difficulties in speaking, problem-solving, and other cognitive skills, along with changes in mood and behavior, which interfere with the person’s daily performance.

Early onset AD is usually termed EOAD, which can be familial (fAD) or non-familial (Nf-EOAD). The fAD develops as a result of rare inherited autosomal dominant genetic mutations predominantly found in amyloid precursor protein (APP)—including Presenilin 1 (PSEN1), and Presenilin 2 (PSEN2)—represent a small percentage (<1%) of the overall AD cases [5]. Symptoms of EOAD develop much earlier than late-onset AD (LOAD) and typically occur before age 65 with some cases as early as age 30. The vast majority of AD cases (>95%) are sporadic late onset (SAD/LOAD) where individuals display obvious symptoms at age 65 or later. LOAD is usually driven by a complex association between age, genetic and environmental risk factors [6]. The uttermost risk factors and causes for AD include age, lifestyle, environmental factors, family history, and bearing the Apolipoprotein E (ApoE) -ε4 gene. Recently, whole-genome sequencing (WGS) studies described rare variants contributing to AD risk (SORL1 and ABCA7, ATP8B4 and ABCA1 and a suggestive signal in ADAM10) [7]. Other rare variants previously described include 29 risk loci implicating 215 immunity processes and microglial activation (i.e., CD33, TREM2, and INPP5D) [8], lipid metabolism [9], tau binding proteins, APP metabolism [10], and four novel loci showing sex-specific association with AD risk (GRID1, RIOK3, MCPH1, ZBTB7C) [11]. Finally, methylation levels at many sites throughout the genome with increasing age that has to be carefully considered when studying AD [12].

The main hallmarks of AD are characterized as two lesions in the brain, namely, the extracellular amyloid plaques and the intracellular neurofibrillary tangles (NFT) [13]. They appear initially in the hippocampus which is responsible for the consolidation of information flow from short-memory to long-memory and extend to the cortical gray matter causing cell death in the brain and compromising their functions. A third pathophysiological marker of AD is a severe loss of neurons in the midbrain cholinergic system that provides the major cholinergic projections to the cortex and hippocampus. Indeed, concurrent inflammatory changes and gliosis together with cerebrovascular disease are contributing factors to AD [14].

During the last three decades, only four drugs have been authorized for AD usage–which help manage some of the symptoms but take no action on the prevention or disease progression [15]. A disease-modifying treatment (DMT) is defined as an intervention that produces an enduring change in the clinical progression of AD by interfering with the underlying pathophysiological mechanisms of the disease process that lead to neuronal death [16]. Consequently, a true DMT cannot be established conclusively based on clinical outcome data alone, such a clinical effect must be accompanied by strong supportive evidence from a biomarker program. In this review, we describe the currently accepted standard of care (SoC) treatment for AD and the most promising strategies for DMT under clinical development: (i) Cholinergic and dopaminergic system and Ca^2+^ signaling, and therapeutical interventions targeting (ii) Amyloid-β protein, (iii) Tau, (iv) inflammation, (v) Lipids and ApoE and (vi) Plasma fractions and Therapeutic plasma exchange. Finally, we critically review current strategies for AD treatment and future areas of improvement.

## 2. Cholinergic and Dopaminergic System and Ca^2+^ Signaling

There is evidence that biological dysfunction or imbalance in neurotransmission, such as the cholinergic and glutamatergic systems, are a key feature in the pathophysiology of AD dementia [17]. Pathological evidence regarding AD shows that degeneration in cholinergic neuron-rich regions is associated with memory loss, agitation, and apathy [18]. Acetylcholine (ACh) has been shown to be highly correlated with memory function, including memory encoding, consolidation storage, and the retrieval process and deficits in both concentration and function of ACh have been found in AD patients. Cholinesterase inhibitors (AChEIs) can improve AD symptoms by preventing synaptic ACh breakdown in the brain. Currently, at least three AChEIs (donepezil, galantamine, and rivastigmine) are approved and being used to treat AD, with some clinical improvement in cognition and global function. However, AChEIs can only improve cognitive symptoms of AD for a certain period but cannot modify the disease course, and benefits are limited, possibly because dosing is restricted by peripheral cholinergic stimulation, which induces several side effects (dose-limiting tolerability issues, including nausea, emesis, diarrhea, muscle cramps, and general malaise). All AChEIs are approved by the Food and Drug Administration (FDA) for mild-to-moderate AD [19]. Over the course of AD, ACh levels decrease due to progressive degradation of pre-synaptic neurons that release ACh into the synaptic cleft. Hence, the efficacy of AChEI decreases with AD progression. Huperzine A, derived from the herb *Huperzia serrata* has a mechanism of action similar to other AChEIs and also has shown to have antioxidant and neuroprotective properties counteracting glutamate-induced toxicity [20]. A meta-analysis of Huperzine efficacy in clinical trials was inconclusive [21].

Another important therapeutic approach involves the use of drugs that act directly on the glutamatergic system, such as memantine. Glutamate is the primary excitatory neurotransmitter in the brain, acting at ionotropic and metabotropic glutamate receptors [22]. Excessive stimulation of glutamatergic signaling results in excitotoxicity, principally mediated by excessive Ca^2+^ entry, primarily through NMDARs causing synaptic dysfunction [23]. Memantine is a noncompetitive NMDAR-antagonist and provides symptomatic treatment of dementia inhibiting the pathological activation of NMDA receptors. Its neuroprotective effects have been demonstrated in several neurological disorders and are used for cognitive disorders in patients with moderate to severe AD.

Additionally, clinical practice guidelines (EMA, NICE) and Scientific Societies recommend the combined use of memantine with AChEIs in advanced stages, being more beneficial than monotherapy with AChEIs [24,25]. Namzaric, which combines memantine and donepezil, received FDA approval in 2014. Since then, most clinical trials have accepted SoC with an AChEI with or without memantine at baseline [26]. Currently, there are no clear recommendations on when to suspend the administration of these drugs, therefore, the interruption of treatment in patients with the advanced disease must be guided and monitored by individualized clinical criteria.

In AD diseased neurons the levels of cytosolic Ca^2+^ are abnormally increased [27]. Increased intracellular cytosolic Ca^2+^ show close relationship with mitochondrial function [28]. Growing evidence in a variety of AD models indicates that calcium dyshomeostasis drastically alters mitochondrial activity which, in turn, initiates the characteristic pathophysiology of AD, including accumulation of Aβ, hyperphosphorylation of Tau, synaptic dysfunction, and neuronal death. On the other hand, neurodegeneration triggered by pathological amyloid-β or Tau requires disturbed Ca^2+^ signaling [29]. Hence, disturbed Ca^2+^ signaling and AD pathophysiology constitute a vicious cycle of mutually reinforcing processes, and, therefore, therapeutic intervention to normalize Ca^2+^ signaling is expected to be therapeutically beneficial. REM0046127 is a small molecule that lowers Orai calcium channel activity and thereby lowers elevated cytosolic Ca^2+^ to physiological levels, but not below. REM0046127 was administered for the first time in humans in the Phase 1 study (NCT04672135) generally safe and well-tolerated [30]. It is predicted that REM0046127 works both as a symptomatic and DMT for AD.

## 3. Amyloid-β Protein

It has been demonstrated that Aβ-amyloid accumulates for 30 years to reach the level typically present in mild AD dementia. Longitudinal studies such as the Australian Imaging, Biomarker, and Lifestyle (AIBL) studies were the first to use prospective analyses to identify how abnormally high levels of Aβ-amyloid (Aβ+), detected via PET were associated with a subtle but relentless decline in memory and other aspects of higher cognition in cognitively normal (CN) older adults [31,32]. Strong genetic and biochemical evidence highlights a central role of the amyloid pathway in the pathogenesis of AD [33,34]. Even though the normal function of APP is not known, it is possibly related to the regulation of neurite outgrowth, cell adhesion, and neuron migration [35]. During the last decade, several studies have described the role of Aβ as an anti-microbial peptide [36]. The amyloid cascade hypothesis is that protein misfolding of the Aβ peptide (due to reduced clearance and/or overproduction) leads to the extracellular accumulation of toxic Aβ aggregates, including β-amyloid plaques, which are the causative factor for the initiation of the neurodegenerative cascade that includes inflammation, gliosis, neuronal damage and synaptic loss [37]. Thus, it has been hypothesized that either suppression of Aβ formation or enhanced clearance will prevent neuronal dysfunction and death.

In the amyloidogenic pathway [38], Aβ is generated from the APP by the sequential proteolysis at the N-terminus of the protein by the β-secretase activity (β-site APP-cleaving enzyme (BACE)) and then processed by γ-secretase complex and secreted from cells of neuronal origin via major regulation as well as a minor constitutive secretory pathway. Aβ is a normal product of cell metabolism and is present in the plasma and in cerebrospinal fluid (CSF) in healthy individuals. The cleavage position of the γ-secretase in the transmembrane domain of APP is imprecise, resulting in the production of Aβ peptides of variable length, which are readily detected in CSF and plasma. Most secreted Aβ is Aβ1–40 but a small component (5–10%) is Aβ1–42 [39], a species that is particularly important in AD. The CSF Aβ42 levels for control groups are ≈900 pg/mL and the plasma Aβ42 levels assayed with IMR are ≈15 pg/mL [40,41]. Aβ species ending at position 42 (Aβ42) aggregate more rapidly than those ending at position 40 (Aβ40) in vitro. In addition, Aβ40 is the main component of amyloid deposition occurring in cerebral amyloid angiopathy (CAA) which has a prevalence of about 50% in patients with AD [42]. It has been shown that CAA contributes to AD dementia independently of senile plaques and neurofibrillary tangles, increase the risk of intracranial hemorrhage and hemorrhagic stroke and is associated with faster rates of cognitive decline. Therefore, CAA pathology may be an important therapeutic target in AD.

**Figure 1:**
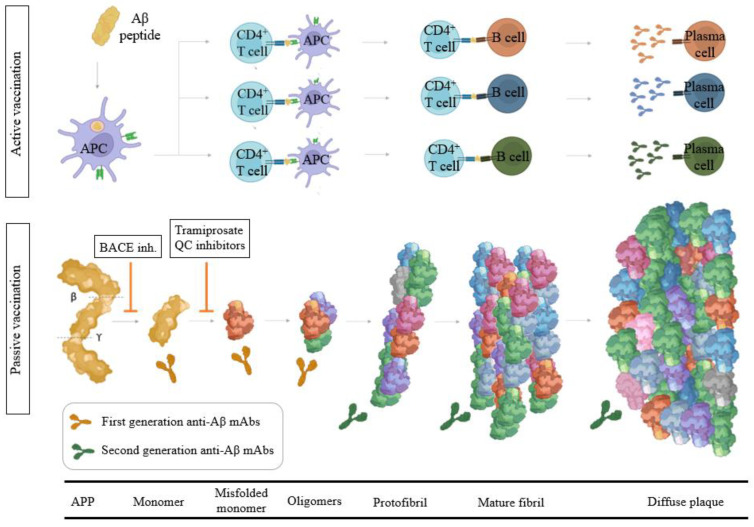
**Anti-Aβ protein strategies for the development of DMT.** Both active and passive immunotherapy are currently under evaluation for reducing amyloid deposition and related progression of cognitive impairment in AD (polyclonal vs. monoclonal approach). In parallel, other small molecules under study target Aβ upstream preventing Aβ misfolding and the generation of toxic peptides.

When these misfolded Aβ reach a critical concentration, oligomers are formed that result in protofibrils and finally culminate in mature fibrils (Figure 1) [43]. Since monomer Aβ and fibrils are in equilibrium, the deposited plaque probably acts as a reservoir for soluble Aβ, and thus eliminating the deposits would have a multifold benefit through the reduced levels of all possible toxic forms of Aβ. The level of soluble, non-fibrillar Aβ oligomers in the brain correlates strongly with the severity of the disease, suggesting that soluble oligomeric species of Aβ, rather than the fibrillary form within amyloid plaques, likely play a pivotal role in AD pathophysiology [44]. Calcium homeostasis perturbation was found to be a ubiquitous toxicity mechanism for soluble oligomers whereas no detectable effect was observed for fibrils [45]. Previous biochemical studies showed that the Aβ deposited in AD brains is heterogeneous, a process known as segmental polymorphism [46]. In a study where Aβ species were quantified, over 90% of the Aβ present ended at Aβ42, and Aβ1–42 comprised only a small fraction of the total Aβ42, indicating that most of the Aβ42 in the AD brain was truncated or modified at its amino-terminus [47]. Prominent examples of these age-modified forms of Aβ include isomerization (isoD-Aβ) and pyroglutamate formation at the N-terminal of Aβ (pGlu-Aβ, pE-Aβ) [48]. IsoD-Aβ, is the result of a chemically spontaneous and non-enzymatic reaction, while the formation of pGlu-Aβ is the consequence of N-terminal truncation by DPP4, followed by dehydration catalyzed by Glutaminyl Cyclase (QC) to form the cyclic pyroglutamate [49]. QC has strong expression in the hippocampus and cortex [50,51]. Brain regions expressing human APP without QC do not display AβpE3 plaque formation. Studies analyzing the amino-terminus of the Aβ in the AD brain showed that Aβ3(pGlu)-42 is an important component of the Aβ deposited in senile plaques of the AD brain, constituting approximately 25% of the total Aβ42 [52]. Aβ3(pGlu)-42 is highly hydrophobic, more prone to oligomerization, and has greater resistance against proteolytic degradation. When compared with full-length Aβ forms, cortical concentrations of AβpE3 correlate more strongly with cognitive impairment and pTau and appear to be more specifically linked to the disease progression [53]. Elevated QC expression in AD tissues has been reported [54], and thus it is tempting to speculate that inhibition of QC might prevent the formation of pGlu-Aβ and suppress downstream pathophysiology [55]. Moreover, AβpE3 is less prevalent in the cerebral vasculature relative to other forms of Aβ and may induce fewer instances of ARIA due to the lower prevalence of AβpE3 in the cerebral vasculature.

### 3.1. Modulators of α-, β-, and γ-Secretase Activity

Blocking the Aβ generation can be attained by blocking the enzymes involved in their production. Despite the fact that it was very appealing for many researchers to develop several inhibitors for γ-secretase, the results of its inhibition were not satisfactory. Transgenic PS1^−/−^ mice were unhealthy, not fertile, and had lagging subventricular areas and cortical dysplasia [56]. Thus, blocking γ-secretase is more likely to result in adverse effects due to the vital biological function of PS1.

BACE1 possesses structural similarities with other aspartyl proteases (such as BACE2, pepsin, renin, cathepsin D, and cathepsin E) [57]. Thus, achieving selectivity in BACE1 inhibition is crucial for developing effective BACE1 inhibitors. BACE1 inhibition provides multiple advantages, among which is the prevention of Aβ formation at an early stage of APP processing. Moreover, BACE1^−/−^ mice exhibited a total loss of Aβ production without any significant side effects [58]. BACE1 inhibitors achieved in humans what they were designed for, namely a dose-dependent lowering of Aβ in the CNS [59]. Importantly, at the time these therapies were developed, a high level of BACE1 inhibition to achieve maximal Aβ lowering (i.e., greater than 60% lowering) was the goal. Several BACE1 inhibitors progressed into late stages of clinical trials all of which unfortunately have been terminated due to the lack of efficacy [60], toxicity (i.e., elevated liver enzymes), and dose-related cognitive worsening. The reason for the absence or even negative effects on cognition is still a matter of debate [61]. One proposed explanation has been that trials were run in too advanced AD populations and tau spreading, and neurodegeneration is already manifested at a point of no return. The umibecestat trial however was run in pre-symptomatic ApoE4 carriers, which is a genetic risk factor for AD associated with increased Aβ aggregate buildup, and still did not meet the endpoint. An explanation could be that inhibition of BACE1 interferes with the processing of the many protein substrates of BACE1, and some of these substrates are related to synaptic plasticity and synaptic homeostasis (i.e., SEZ6 and NRG1). All this clinical trial data suggest on-target toxicity is likely a contributing factor, which implies the only potential future of BACE1 inhibitors lies in a careful titration of highly selective compounds in early populations where the amyloid burden is still minimal as prophylactic therapy, or as affordable oral maintenance therapy following amyloid-clearing therapies with the goal of to achieve a low level of BACE1 inhibition (i.e., Aβ lowering 15–30%) [62]. Support for this hypothesis can be found indirectly in the protective A673T APP mutation that leads to only 25% reduced Aβ production and still avoids carriers getting AD [63]. It should be noted that these mutations lead to a reduction in Aβ production from birth onwards, which will be difficult to reproduce via pharmacological treatment.

### 3.2. Aβ Immunotherapy

Active vaccination with Aβ1–42 was first described by Schenk et al. in 1999 [64]. Their report demonstrated that active immunization with Aβ1–42 in the PDAPP transgenic mouse reduced levels of Aβ deposits dramatically and protected mice from memory deficits. Since then, several studies have shown that both active and passive immunotherapy were effective in reducing amyloid deposition in transgenic mice models when performed as a preventative measure; however, when these approaches are performed in aged transgenic mice with pre-existing deposits, they showed diminished or no efficacy. Three main mechanisms of action for Aβ immunotherapy have been postulated: soluble equilibrium, phagocytosis, or blockade of amyloid seeding [65]. The soluble equilibrium mechanism is based upon antibodies neutralizing soluble Aβ and shifting the equilibrium to favor dissolution. This mechanism of action is proposed to take place in both the periphery and central compartments. The phagocytosis mechanism requires antibodies to gain access to the CNS, where they engage deposited amyloid and facilitate microglial-mediated phagocytosis of the plaque. Finally, others have postulated that the prevention of amyloid deposition may be due to antibodies binding to early amyloid seeds at a point in the cascade when these species are present at low abundance, thus preventing amyloid propagation.

#### 3.2.1. Aβ Passive Immunotherapy

Passive immunotherapy involves the direct administration of external antibodies. Intravenous immunoglobulin (IVIG) products were first investigated as possible therapeutic agents for MCI and AD but disappointing results were obtained in Phase 2/3 AD trials [66,67]. Aβ monoclonal antibodies (mAbs) in development target different epitopes and vary in their detection and binding affinity to several Aβ species. High doses of antibodies in the periphery are required because of the low-level (0.1–0.3%) penetration across the blood-brain barrier (BBB) to effectively engage the local (i.e., CNS) mechanisms for clearing the cerebral amyloid [68].

First-generation anti-Aβ antibody-based immunotherapy drugs were terminated in clinical trials due to the lack of cognitive benefits for AD patients (Figure 1). It is hypothesized that the inability of the N-terminal antibodies to remove existing plaque was due to antibody saturation with soluble Aβ upon entering the CNS. Bapineuzumab was administered at a maximal dose of 2 mg/kg (showing 14% ARIA-E) [69]. This mAb does not bind to truncated Aβ species as it recognizes the 1–5 of the Aβ epitope. Solanezumab, a humanized monoclonal IgG1 antibody of m266, with epitope 16–26 targeting soluble Aβ species (this epitope is buried in aggregated forms) and exhibited strong binding to monomers of Aβ40 or Aβ42 and also inhibited primary nucleation [70]. Phase 3 Expedition 3 clinical trial with solanezumab at 400 mg monthly infusions showed an inability to significantly reduce amyloid cortical burden (with no related ARIA-E or ARIA-H) [71]. In June 2017, the A4 study was initiated by quadrupling the dose from 400 to 1600 mg in symptomatic brain amyloid deposition. The trial will run until the end of 2022 (NCT02008357). Crenezumab is a humanized anti-Aβ IgG4 against the 13–24 Aβ epitope that binds monomeric and aggregated forms of Aβ, with the highest affinity for soluble oligomers, and it can block aggregation of monomers and induce disaggregation of existing Aβ aggregates in vitro. Effects were evaluated in ABBY (NCT01343966) and BLAZE (NCT01397578) Phase 2 trials with mild-AD patients were a dose up to 60–120 mg/kg were evaluated [72]. AβO levels were significantly decreased and Aβ42 monomer levels were increased in cerebrospinal fluid but PET amyloid load was not lowered [73].

Encouragingly, recent studies using second-generation mAbs that target highly specific epitopes against aggregated Aβ have shown substantial reductions in PET amyloid, with many participants becoming amyloid negative by 6–14 months of high dose treatment, showing that some indication of slowing cognitive decline can be achieved with anti-amyloid immunotherapy (Figure 1) [74]. These mAbs acts centrally recruiting microglia and also, reducing secondary nucleation. Aducanumab, (Aduhelm, BIIB037) on 7 June 2021, become the first DMT approved by the FDA for the treatment of early AD [75,76]. Aducanumab is a high-affinity, fully human IgG1 mAb against Aβ epitope 3–7 and binds to soluble Aβ aggregates and insoluble fibrils with >10,000-fold selectivity over monomers and also has demonstrated inhibition of secondary nucleation. Amyloid deposition was reduced in all treatment groups at 10 mg/kg during 26 weeks [77,78], 41.3% in the combined 10 mg/kg aducanumab group (*n* = 1029) experienced ARIA [79]. Lecanemab (BAN2401) is the humanized IgG1 version of the mouse mAb158, which selectively binds to large, soluble Aβ protofibrils. The antibody administered at a dose of 10 mg/kg bi-weekly reduced brain amyloid by up to 93 percent in the highest-dose group [80]. This dose slowed cognitive decline by 47 percent on the ADAS-Cog, and by 30 percent on the ADCOMS. Donanemab is a plaque-specific antibody that targets Aβp3–42, which showed rapid—a six-month course of 20 mg/kg-and robust clearance of pre-existing plaque slowing decline on the iADRS by 32 percent compared to placebo at 18 months and 27% of patients treated with the mAb developed ARIA-E [81]. Donanemab is a humanized IgG1 antibody developed from the murine IgG2a antibody mE8. Donanemab is itself strongly immunogenic and 90% of patients who received the mAb mounted an immune response against it (presence of anti-drug antibodies, ADA, which could potentially impact pharmacokinetics). Gantenerumab is a fully human IgG1 antibody designed to bind with subnanomolar affinity to Aβ fibrils binding two discontiguous regions of Aβ, with the highest affinity at residues 2–11 and 18–27 (residues 3–11 and 18–27, which are hidden within most fibrils). Two years of high-dose (1020 mg/month) subcutaneous administration of higher doses of gantenerumab in the SCarlet and Marguerite RoAD extension studies lowered brain amyloid by an average of 59 centiloid, opening the possibility of at-home administration. Novel strategies are currently under development to increase the BBB penetrance of mAbs. RG6102 is a hybrid molecule that consists of the Fc portion of gantenerumab, i.e., its tail, conjugated to the Fab shuttle that binds to transferrin. In mice, a mouse version of RG6102 entered the brain in 12-fold higher quantities than gantenerumab and cleared plaque at lower doses [82,83]. The brain shuttle-gantenerumab CSF/plasma ratio was 0.8% becoming the first evidence of a brain-shuttling effect on the CNS compartment in humans. Lecanemab (NCT03887455 and NCT04468659), Gantenerumab (NCT03444870), and Donanemab (NCT05026866) are currently under Phase 3 of clinical development and FDA has granted breakthrough therapy designation [84], and different Phase 3 head-to-head trials comparing anti-Aβ mAbs to assess superiority of brain amyloid plaque clearance are ongoing (NCT05108922).

However, passive immunotherapy trials have been associated with the highly frequent occurrence of Amyloid Related Imaging Abnormalities (ARIA), referring to a spectrum of imaging abnormalities detected on MRI scans suggestive of ARIA-E or ARIA-H corresponding to microhaemorrhages and hemosiderin deposits and are associated with an accumulation of amyloid in the cerebrovasculature [85,86]. Interestingly, prolonged intracerebroventricular (icv) delivery of anti-Aβ antibodies dose-dependently reduced the parenchymal plaque burden, astrogliosis, and dystrophic neurites at doses 10- to 50-fold lower than used with systemic delivery of the same antibody in an aged Tg2576 mouse model of AD and icv-infused antibodies reduced CAA and associated micro-hemorrhages compared with systemically delivered anti-Aβ mAbs [87].

#### 3.2.2. Aβ Active Immunotherapy

Active vaccination is defined as introducing an exogenous substance to stimulate the immune system to mount a response (Figure 1) [88]. The multitargeted profile of the polyclonal antibodies generated by active vaccines may improve their probability of success in patients at different AD pathological stages with regard to single-target mAbs [89].

Active immunization with aggregated full-length Aβ1–42 (AN1792) associated with a Th1 adjuvant, was the first immunotherapy tested in AD patients, which generated anti-Aβ antibody responses in 25% of patients and showed decreased level of tau protein in the CSF and demonstrated some clinical benefit. In this trial [90], 18 patients out of 298 given the AN-1792 vaccine developed treatment-related meningoencephalitis likely caused by autoreactive T-cell activation and Aβ-reactive T-cell infiltration into the CNS and the sponsor suspended the trial [91,92].

Several second-generation Aβ-targeting vaccines have been subsequently designed to minimize Aβ-related T-cell inflammation including: ACC-001 using Aβ1–7 peptide conjugated to diphtheria toxoid protein [93], CAD106 using Aβ1–6 peptide coupled to Qb virus-like particle, V950 using multivalent Aβ1–15, and affitopes AD01 and AD02 using Aβ mimetics conjugated to KLH [94,95]. CAD106 (Generation Study 1, NCT02565511) to treat individuals with the ApoE4 allele and amyloid burden without cognitive impairment is ongoing [96]. In animals, CAD106 induced Aβ-antibody titers without activating Aβ-reactive T cells. CAD106, currently in clinical Phase 3 trial, has completed two Phase 2 trials reporting acceptable safety and evoking strong serological responses in 80% of patients [97]. ACI-24 is a liposome vaccine-based array of Aβ1–15 sequences, anchored into the surface of liposomes in such a way that the peptides adopt an aggregated β-sheet structure, forming a conformational epitope [98]. UB-311 is a mixture of 2 synthetic peptides having active UBITh^®^ helper T-cell epitopes and B-cell epitopes from the first 14 amino acids of the N-terminus of amyloid beta (Aβ1–14) [99]. In the Phase 1 study (Study V118-AD), UB-311 elicited antibodies with specificity to the Aβ1–14 domain in all participants. ABvac40 comprises multiple repeats of Aβ33–40 using the carrier protein KLH. Unlike N-terminal end Aβ-directed antibodies, anti-C-terminal end Aβ antibodies do not bind to the unprocessed protein, preventing the accumulation of potentially toxic antigen-antibody complexes around neurons and could provide protection against N-terminally truncated Aβ peptides [100]. Indeed, 88% of the patients receiving the vaccine showed specific anti-Aβ40 antibodies that recognized monomeric, oligomeric, and insoluble (plaques) forms of the Aβ40 peptide.

To date, these and related vaccines have not presented convincing Aβ brain removal or clinical efficacy data. ARIA are less frequent after active anti-Aβ immunization. The number of immunizations and antibody titer should be optimized in each case due to the different stability of the peptides used for immunization and the immunogenicity of such peptides together with the reduced immune system response in elderly population.

### 3.3. Modulators of Aβ Toxicity

TMEM97 (recently named Sigma-2 receptor) is highly found in synapses and interacts with Aβ. The TMEM97 complex allosteric antagonist CT1812 blocked the formation of the TMEM97-Aβ complex [101]. These data obtained in experimental models of AD support a role for TMEM97 in the synaptic binding of Aβ in AD where it may mediate synaptotoxicity through the modulation of intracellular Ca^2+^ levels. CT1812 is neuroprotective and reduces cognitive deficits and neuroinflammation [102].

Tramiprosate, and its prodrug ALZ-801 showed significant clinical effects in the homozygous for ApoE4 [103]. A novel multi-ligand enveloping stabilizing effect of the small molecule Tramiprosate, modulates conformational flexibility of Aβ and stabilizes Aβ42 monomers resulting in the prevention of Aβ-oligomer formation [104]. Reductions in p-Tau levels and dose-dependent preservation of hippocampal volume have been observed in the ApoE 4/4 population and a Phase 3 in this population is currently ongoing (NCT04770220) [105].

Pharmacological QC inhibition reduces pGlu-Aβ levels and cerebral amyloid burden, improving cognitive function in transgenic AD mouse models. To date, the only QC inhibitor in clinical trials is PQ912 [106]. Recently, a randomized, double-blind, placebo-controlled Phase 2a SAPHIR trial to evaluate doses of PQ912 for 3 months in MCI or early AD showed significantly reduced YKL-40 and neurogranin compared with the placebo group, brain electrical rhythms were normalized and benefits on working memory were observed [107]. QC activity is responsible for the conversion of monocyte chemotactic proteins to their bioactive pGlu-modified forms, and inhibition by the QC inhibitor PBD150 reduces monocyte migration. Thus, the contribution of QC activity to AD pathology may therefore be multi-faceted. Intriguingly, PBD150 reduces pGlu-Aβ levels and total amyloid burden in the brains of transgenic AD mouse models, despite its reported inability to cross the murine BBB [108,109]. A blockade of AβpE3 formation by inhibiting QC may cause uncertain side effects, given that QC can process the N-terminal of many other subtracts.

## 4. Tau

The interaction of Aβ and tau in the pathogenesis of AD is a subject of intense inquiry, with the bulk of evidence indicating that changes in tau are downstream of Aβ [110,111]. Aβ and tau pathologies initially proceed independently but at a certain point in the progression of AD, the Aβ is involved in the alteration of tau. Tau protein is a member of the family of microtubule-associated proteins (MAP) and is essential for microtubule assembly and stabilization in neuronal cells. Tau protein is composed of four regions: an N-terminal projection region, a proline-rich region (PRR), a microtubule-binding region, and a C-terminal region (CTR) [112]. Under physiological conditions, tau associates with microtubules in neuronal axons. The majority of extracellular tau in human CSF is monomeric and C-terminal truncated [113,114]. Extracellular tau (eTau) may mediate tau spreading and serve as a biomarker for AD. The source of extracellular tau protein may also be independent of cell death or neurodegeneration. In agreement, it has been described that propagation of tau pathology occurs trans-synaptically in a prion-like mechanism [115,116]. NFTs containing paired helical filaments (PHFs) are cytoplasmic filaments comprising aggregates of abnormally hyperphosphorylated tau proteins [117]. Hyperphosphorylated Tau is known to dissociate from microtubules. Given that tau pathology correlates better with cognitive impairments than Aβ lesions, targeting tau is expected to be more effective than Aβ clearance once the clinical symptoms are evident.

### 4.1. Tau Passive Immunotherapy

**Figure 2:**
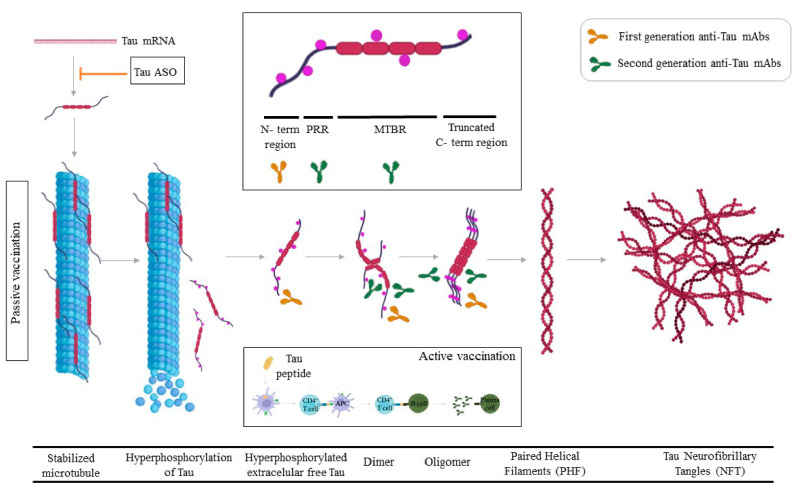
**Anti-Tau strategies for the development of DMT.** Passive immunotherapy is the main strategy under evaluation for reducing the cell-to-cell spread of extracellular Tau. At this moment specific phosphor-Tau mAbs are under clinical development. Other strategies such as active vaccination and antisense oligonucleotides to reduce Tau levels are also under evaluation in clinical trials.

The rationale for this therapeutic approach is that eTau is proposed to be involved in the spread of pathology in tauopathies. The first generation of anti-Tau mAbs was directed against the N-terminal region of Tau (Figure 2). Gosuranemab (BIIB092) is a humanized IgG4 anti-tau mAb against eTau. Binding experiments showed that gosuranemab exhibited a high affinity for tau monomer, tau fibrils, and insoluble tau, and results from clinical trials confirmed that gosuranemab reduced CSF unbound N-terminal tau fragments by 98% [118]. A Phase 2 Study of BIIB092 in participants with Early Alzheimer’s Disease (NCT03352557) was stopped based on lack of efficacy, and not because of any safety concerns. Tilavonemab recognizes an aggregated, extracellular form of pathological tau, binding to tau’s N-terminus. Despite a confirmed decrease in CSF-free tau the studies in subjects with Early AD (NCT02880956) and Progressive Supranuclear Palsy (PSP) have been stopped based on lack of efficacy [119,120]. Semorinemab, a mAb that binds to the N-terminus 6–23aa of all six isoforms of human tau, both monomeric and oligomeric, regardless of phosphorylation status, has recently shown (31 August 2021), results indicating a 43.6% slowing of decline on the ADAS-Cog11 co-primary, which enrolled participants in the moderate stages of AD [121]. In a previous Phase 2 study, called TAURIEL, semorinemab brought no cognitive or functional benefit to people with Early AD. These results demonstrate that mAbs anti-Tau could be useful in more advanced stages of AD compared with anti-Aβ mAbs.

Several different P-tau species can be quantified in CSF and plasma, including P-tau181 and P-tau217, and recently also other species such as P-tau231 and P-tau235 and plasma P-tau accurately predict progression to AD dementia in individuals with memory complaints [122,123]. Currently, the second generation of anti-Tau mAbs is under clinical development including phopho-specific anti-Tau mAbs (i.e., PNT001/NCT04677829 and JNJ-63733657/NCT04619420 that bind to p-Tau231 and p-Tau217, respectively) [124,125], mid-region anti-Tau mAbs (i.e., Bepranemab/ NCT04867616) or early pathological forms of Tau (Zagotenemab/LY3303560 against a pathological form of soluble Tau which forms before the assembly of PHF) are under clinical development in patients with early AD (Figure 2) [126]. In parallel, novel strategies to downregulate Tau such as antisense oligonucleotide (ASO) are under study (NCT03186989).

### 4.2. Active Tau Immunotherapy

ACI-35.030 vaccine is designed to elicit antibodies against phosphorylated Tau protein. The vaccine contains a synthetic tau fragment phosphorylated at residues S396 and S404 anchored into a lipid bilayer [127]. A Phase 1b/2a (NCT04445831) is currently running and all participants presented antibodies preferentially against phosphorylated tau.

AADvac1 is a therapeutic vaccine derived from amino acids 294 to 305 that targets misfolded tau protein, coupled to KLH and aluminum hydroxide. After the first six doses, 96.5% of participants in the treatment group produced detectable levels of IgG [128]. Plasma NfL increased in both groups over the 104-week trial, but less in the vaccine group (27.7% in the placebo vs. 12.6% in the active group), and no changes in cognitive decline were observed [129].

## 5. Inflammation and AD

**Figure 3:**
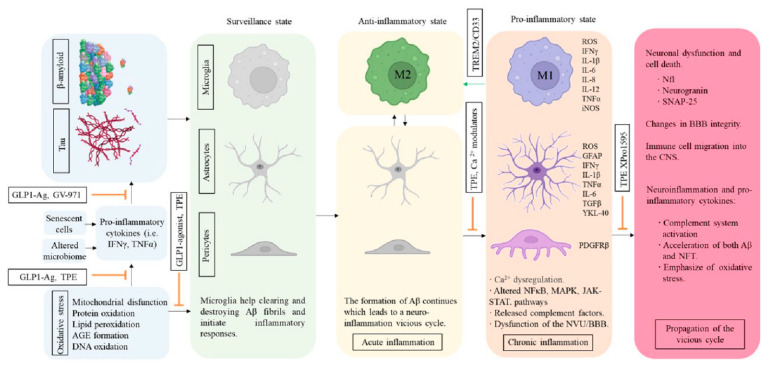
**Anti-inflammatory strategies for the development of DMT.** Inflammation plays a central role in AD and at this moment is the main therapeutical strategy in clinical development for AD. Some molecules target specific players of inflammation or microglial activation (such as TNFα, TREM2, or CD33) while others have a broader anti-inflammatory effect (GLP1 agonist, Plasma exchange).

Recent data suggest that neuroinflammation is present in the brain prior to cognitive decline and that Aβ deposits are associated with inflammatory proteins and microglia in the early stages of AD pathology [130]. A significant number of pro-inflammatory molecules involved in CNS diseases have been identified, such as interleukin 6 (IL-6), tumor necrosis factor (TNFα), and the inflammasome complex (NRLP3) and circulating levels of peripheral cytokines have been shown to correlate to cytokine levels in the brain, AD severity, brain atrophy, and cognitive performance (i.e., TNFα, MCP-1) [131,132,133]. Neuroinflammation can both harm and help the AD brain [134]. Some studies suggest a two-peak hypothesis of inflammatory activity in AD, and the first peak being driven by amyloid aggregation and possibly having a protective role and the second being driven by tau tangle formation and being neurotoxic [135]. Then, activation of the innate immune response is initially likely to be beneficial [136]. However, long-term innate immune activation causes chronic proinflammatory conditions and the release of endogenous factors (Aβ, calprotectin, proinflammatory cytokines, complement activation, and tau hyperphosphorylation) that can drive destructive cascades (Figure 3) [137]. Several studies have shown that both CSF and plasma YKL-40 levels are higher in AD patients compared with controls [138,139]. The activation of microglia and astrocytes as a reaction to the ongoing deposition of Aβ and NFTs triggers the production of several proinflammatory signal molecules including cytokines, chemokines, complement molecules, growth factors, and cell adhesion molecules. Previous studies suggest that microglia transition from a largely anti-inflammatory/pro-phagocytic (M2) to a pro-inflammatory/neurotoxic (M1) activation state during AD progression [140]. Profiling of microglia transcriptome in mouse models of Aβ accumulation and AD patients has revealed a robust transcriptional activation signature which has been referred to as disease-associated microglia (DAM), which is quite distinct from that of homeostatic microglia [141]. Previously, many scientists thought that dialing down inflammation would help, but growing evidence now suggests the opposite. Single nucleotide polymorphisms (SNPs) and/or differential expression of microglial receptors such as TREM2, CD33, ApoE, and ABCA7 have been strongly associated with an increased risk of developing AD [142].

### 5.1. Modulators of Microglial Activity and Neuroinflammation

Several epidemiological and observational studies have indicated that exposure to nonsteroidal anti-inflammatory drugs (NSAIDs) protects against AD [143]. In preclinical models, Dexibuprofen (DXI), improved insulin resistance, activated the non-amyloidogenic pathway, and improved cognition [144,145]. However, the lack of efficacy of naproxen against AD in clinical trials (NCT00004845, NCT02702817) in participants with mild-to-moderate AD suggested that more specific inflammation mechanisms must be identified to inhibit AD-related neuroinflammation [146,147]. Cromolyn sodium, a small molecule approved for the treatment of asthma (with structural similarity to fisetin, an anti-Aβ aggregation molecule) [148] reduced Aβ levels in transgenic AD mouse brains and showed increased Aβ42 uptake in microglial cell-based assays [149,150]. Cromolyn is being evaluated in Phase 3 clinical trials (NCT02547818) in early AD patients. Other interesting molecules targeting inflammation are Etanercept (FDA-approved for the treatment of rheumatological and inflammatory skin conditions) and XPro1595, both directed against TNFα [151,152]. XPro1595 is a pegylated protein biologic that targets and neutralizes the inflammatory cytokine TNFα forming heterotrimers with native soluble TNFα and preventing its interaction with the type 1 TNFα receptor [153]. Etanercept and XPro1595 are currently under evaluation in patients with inflammatory biomarkers (NCT01068353, NCT05321498) [154].

Microglial cells dynamically survey the environment; they are responsible for the elimination of pathogens, cellular debris, dead cells, remodeling synapses, and the clearance of toxic proteins. They are partly dependent on the colony-stimulating factor-1 receptor (CSF1R) signaling for their maintenance, activation, proliferation, and self-renewal [155]. Data obtained from oral PLX5622 treatment, a highly selective brain penetrant CSF1R inhibitor, or studies using CSF1R^−/−^ mice provided clear evidence that deletion of CSF1R prevents the accumulation and/or induced a better clearance of Aβ in the brain accompanied by CAA onset [156,157]. Thus, the absence of microglia modulated the location of Aβ accumulation. The inhibition of CSF1R would not only affect microglia but also other populations (monocytes, macrophages, and osteoclasts), possibly causing an immunosuppressive effect [158].

Triggering receptor expressed on myeloid cells 2 (TREM2) expressed by many cells of the myeloid lineage, including microglial cells) regulates myeloid cell number, phagocytosis, and inflammation [159]. Increased expression of TREM2 on microglia is coupled with enhanced phagocytic pathways and promotion of the alternative activation state of microglia, which is thought to be protective. The other signaling stream suppresses inflammatory reactivity and involves the repression of cytokine production and secretion. TREM2 expression promotes microglia survival and proliferation by transmitting intracellular activating signals through the adaptor DAP12 [160]. The R47H partial loss-of-function variant of human TREM2 impairs ligand binding and increases a person’s risk of developing AD [161]. In AD mouse models, defective TREM2 function exacerbates Aβ accumulation tissue, whereas TREM2 overexpression attenuates pathology [162]. Thus, AD may benefit from TREM2 activation. Some anti-TREM2 antibodies claim to act as agonistic antibodies, activate the receptor, and stimulate microglia to remove amyloid (Figure 3). In cell culture, AL002a treatment increased phosphorylation of Syk, a downstream effector of TREM2 signaling [163]. Interestingly, mutations of the Fc region of AL002c that block binding to Fc receptors and complement only slightly quelled its pro-survival effect on myeloid cells, suggesting that AL002c-mediated activation of hTREM2 function is largely independent of mAb-mediated cross-linking of Fc receptors. AL002a into 5XFAD mice intraperitoneally doubled the number of microglial cells surrounding amyloid plaques and halved the amount of Aβ and improved behavior [163]. These results suggested that injection of AL002c expands microglia transitioning from homeostatic to proliferating microglia and shifts the microglial phenotype from M1 to M2 via the TREM2. mAb 4D9 increases the full-length TREM2 on the cell surface both stabilizing TREM2 on the cell surface and reducing its shedding (reduction of sTREM2) [164].

CD33 is primarily expressed in cells of the myeloid lineage, especially monocytes and dendritic cells [165]. In the brain, CD33 is exclusively expressed by microglia and infiltrating macrophages. CD33 expression is elevated in AD brains, where it is thought to modulate microglial activation and inhibit Aβ microglial uptake and clearance. CD33 is an inhibitory receptor and opposes the effects of TREM2 signaling (Figure 3). Studies using CD33^−/−^ mouse models resulted in lower Aβ levels and reduced amyloid plaque [166]. This supports the notion that reduced expression of CD33 allows more efficient phagocytic clearance of pathogenic Aβ by microglia and thus protects against AD [167]. Since the protective allele of CD33 is associated with reduced CD33 expression, it is relatively clear that the therapeutic approach for targeting CD33 would be inhibition of its activity via lowering levels of the total or active/functional form of the protein [168]. Notably, two CD33 antibodies, gemtuzumab ozogamicin (Mylotarg) and lintuzumab, have been tested in humans for treating acute myeloid leukemia and can effectively reduce cell surface CD33 expression in monocytes [169]. The CD33 antibodies lower CD33 protein levels mostly by inducing internalization and degradation or by inhibiting CD33 activity and they have been repurposed for treating AD [170].

Finally, GV-971, a mixture of acidic linear oligosaccharides derived from brown algae and Ginkgo Biloba leaf extract, has been described to improve inflammation in AD. GV-971 is proposed to inhibit systemic and neuroinflammation by promoting changes in the gut microbiome [171]. In a Phase II study, a significant drug-placebo difference in the ADAS-Cog12 favoring GV-971 was present [172]. Ginkgo Biloba has been related to an anti-inflammatory and antioxidant effect [173]. Furthermore, Ginkgo Biloba manifested a beneficial effect on the circulatory system (blood flow improvement, reduced platelet aggregation, reinforcing the walls of the capillaries), reducing one of the most important comorbidities associated with AD [174].

### 5.2. Cerebrovascular Structure and Insulin Resistance

Chronic inflammation is a common denominator of neurodegenerative diseases and generates dysfunction of the BBB and an inflammatory stimulus [175,176]. The brain has little energy reserve and requires a continuous supply of glucose and O_2_ through cerebral blood flow (CBF). Aβ can deposit and interfere with the vascular milieu, exert a toxic effect, induce vascular inflammation, and contribute to vascular pathology [177]. Cerebrovascular structure and cerebrovascular function are also altered in AD. Furthermore, insufficient CBF may alter Aβ trafficking across the BBB [178].

Capillary pericytes are of crucial importance in regulating diverse microvascular functions in numerous CNS disorders including AD [179]. In these disorders, pericyte malfunction often leads to BBB disruption and/or a decrease of CBF, thus causing secondary neurological damage [180]. Platelet-derived growth factor receptor β (PDGFRβ) is expressed in the brain mainly in brain capillary pericytes and levels of soluble PDGFRβ (sPDGFRβ) could be used as a biomarker of pericyte injury, as elevated sPDGFRβ levels in biofluids have been measured in patients with dementia [181]. Aβ oligomers constrict human capillaries in AD via signaling to pericytes [182]. These changes suggest that therapies aimed at maintenance of normal pericyte function in AD may serve to preserve neuronal function longer [183,184]. Small rises in cytoplasmic Ca^2+^ in pericytes amplify capillary constriction [185]. A potential therapeutic approach would be to apply blockers of pericyte voltage-gated Ca^2+^ channels. Endothelial cells release PDGF-BB which binds to PDGFRβ on pericytes, enhancing their proliferation and recruiting them to the endothelial tube [186]. Icv administration of exogenous PDGF-BB has entered human clinical trials (NCT02408562) and appears to be well-tolerated and safe.

In several studies, type II diabetes mellitus (T2DM) has been identified as a risk factor for AD [187]. Meta-analyses indicate that diabetes increases the risk of all-cause dementia by a factor of 1.51, VaD by a factor of 2.48, and AD by a factor of 1.46 [188]. It has been proposed that AD is type 3 diabetes mellitus because many features of insulin resistance are also visible in AD (inactivation of IGF-1 and IRS1/2) [189]. While insulin desensitization in diabetes is driven by high glucose and insulin levels, insulin desensitization in the brain of AD patients is most likely driven by chronic inflammation. Apart from controlling blood glucose, insulin has the general physiological profile of a growth factor (insulin modulates neuronal development, synaptic transmission, and neuroprotection, whereas insulin resistance impairs learning and memory and increases neurodegenerative disease risk) [190]. Pro-inflammatory cytokines such as TNF will block growth factor signaling such as that of insulin or IGF-1 [190]. Disturbed insulin signaling is a mechanism by which soluble Aβ modulates tau phosphorylation, whereas Aβ oligomers induce both tau phosphorylation and IRS-1 inactivation raising the possibility of a pathogenic feed-forward loop [191]. A randomized, double-blind, and placebo-controlled clinical trial (NCT01767909), evaluated in either MCI or mild to moderate AD the effect of intranasal insulin showing that treatment with insulin improved memory and functional abilities [192]. Insulin is not an ideal drug to be developed as a major treatment for AD and PD, as higher insulin levels progress insulin desensitization further [193,194]. Incretin hormones GLP-1 and GIP drugs do not enhance insulin desensitization as they do not activate insulin receptors [195]. Instead, they can re-sensitize insulin signaling and do not affect blood glucose levels in normoglycemic people. Pooled data from three randomized controlled cardiovascular outcome trials showed promising results for repurposing GLP-1 agonist as a treatment for AD [196]. The side effects include nausea and loss of appetite [197].

GLP-1 is part of the peptide growth-factor family and activates a G-protein coupled receptor (GPCR) expressed in neurons with comparable downstream signaling effects to those of insulin [198]. GLP-1 receptor agonists such as liraglutide, lixisenatide, and semaglutide are on the market, already licensed to treat T2DM and obesity in the EU and other countries, have neuroprotective and anti-inflammatory effects in animal models of AD and PD and re-sensitize insulin signaling [199]. GLP-1 receptor agonists can cross the BBB [200] and its metabolite inactive GLP-1 9–36 is neuroprotective [201]. Recently, a randomized 12 month in mild to moderate AD patients treated with liraglutide (NCT01843075) showed that GLP1 analogs can improve cognitive function and MRI volume in AD subjects [202]. At this moment, different GLP1 agonists (i.e., liraglutide, semaglutide) are being evaluated for AD in different subcutaneous and oral formulations in MCI and mild AD [203]. GIP is a 42 amino acid long peptide hormone expressed in neurons. The GIP receptor is a GPCR with similar protective properties as GLP-1 [204]. As GIP and GLP-1 both have their protective effects and work together in cell signaling, novel GLP-1 and GIP receptor dual agonists are being developed and tested in clinical trials in patients with T2DM and show superior performance compared to liraglutide [205].

## 6. Lipids and ApoE

The most common genetic risk factor of AD is the ApoE4 genotype [206]. ApoE has multiple functions and plays key roles in lipid metabolism and neurobiology [207]. Its major function is to transport lipids among various cells and tissues of the body. Disruption of lipid homeostasis is related to neurodegenerative diseases such as AD [208]. Lipids are at the center of AD pathology based on their involvement in the BBB function, APP processing, myelination, membrane remodeling, receptor signaling, inflammation, oxidation, and energy balance.

Statins are among the most frequently prescribed medications [209]. These medications have a host of pleiotropic effects and assist to inhibit the inflammation process, improve vascular flow, and suppress the production of reactive oxygen species [210]. The benefits linked with these medications are very important in fighting cardiovascular disease. Statins lower low-density lipoprotein cholesterol and triglycerides while increasing high-density lipoprotein cholesterol. Several preclinical studies using mice showed that simvastatin or fluvastatin attenuated oxidative stress and inflammation in the APP, mitigate oxidative damage, and ameliorate neural degeneration and cognitive dysfunction [211]. Statins (i.e., batimastat, marimastat, simvastatin, atorvastatin) are well-known cholesterol-lowering drugs that have been suggested to regulate the α-secretase resulting in anti-AD effects [212,213]. However, evidence for this effect has not been consistent between different observational studies, statins appear modestly to reduce the risk of developing AD. Simvastatin had no benefit on the progression of symptoms in mild to moderate AD patients (NCT00053599).

ApoE plays a role in Aβ metabolism and clearance, in which ApoE4 is the least efficient variant. Different studies analyzing ApoE-Aβ interaction provide strong support to the concept that decreasing the levels of ApoE4 specifically will have a therapeutic implication [214]. The first approaches for this therapeutic strategy were evaluated using an anti-ApoE4 mAb 9D11 that binds specifically to ApoE4 [215]. Direct icv application of mAb 9D11 prevented accumulation of Aβ in hippocampal neurons and Tau hyperphosphorylation and was associated with reversal of the cognitive impairments [216]. Another Anti-ApoE mAb HAE-4 cleared plaque from a mouse model and also cleared amyloid deposits from blood vessel walls [217]. Notably, HAE-4 did all this without causing microhemorrhages, hinting that this approach might be safer than using anti-Aβ mAbs.

In 2018, the first study to convert ApoE4 to the protective ApoE2 started [218]. NCT03634007 is a one-time ascending dose open-label Phase 1/2 study designed to evaluate gene therapy to treat ApoE4 homozygotes patients. The study assessed the safety and toxicity of intrathecal administration of LX1001 (an adeno-associated virus gene transfer vector expressing the cDNA coding for human ApoE2) [219]. Findings in the first patient group treated showed increased ApoE2 levels and reduced tau protein in CSF. Recently, LX1001 has been granted fast-track designation by the U.S. FDA.

## 7. Plasma Fractions and Therapeutic Plasma Exchange

Two parallel processes occur simultaneously in our body during aging/AD: (i) products of pathophysiological processes in the AD brain are shown to diffuse to the blood and (ii) different waves of changes in the proteome appear during aging [220]. This new approach to the study of aging led to the identification of unexpected signatures and pathways that might offer potential targets for age-related diseases. The investigation of plasma fractions in AD is also supported by the theory that there are specific components of plasma that actually drive beneficial functions in aging.

The hypothesis that removal of toxic and senescent factors from blood and simultaneously supplementing “rejuvenating” elements may be beneficial in aging and in AD has been quite extensively tested in animals (parabiosis, serum-injections, probiotics, fecal microbiota transplantation) [221]. The effects seen by animal models, such as heterochronic parabiosis, which consist of the surgical joining of two animals of different ages, included the rejuvenation of multiple tissues in the old partner stimulating neurogenesis, synaptic plasticity in the hippocampus, and improved cognitive function [222]. In preclinical studies, administration of plasma from young healthy mice to AD transgenic mice improved cognitive deficits without affecting brain amyloid plaques [223]. Microglial and astrocytes “rejuvenation”, via peripheral manipulation of the hematopoietic system, may be sufficient to maintain or restore hippocampal function [224].

Therapeutic plasma exchange (TPE), consisting of removing blood plasma and exchanging it with donated blood products, is widely used in the treatment of various pathologies of the CNS (i.e., Guillain-Barré syndrome, multiple sclerosis, and acute inflammatory demyelinating disease), and has been proposed for treating AD to remove senescent or toxic factors from the periphery [225]. The initial rationale for TPE in AD was that the vast majority of Aβ circulating in the blood (around 90%) circulates bound to albumin in a 1:1 ratio and TPE could remove the excess of Aβ from the brain and provide clinical benefits by altering Aβ equilibrium between brain and plasma [226]. TPE is used to remove the patient’s plasma and replace it with albumin or other colloids while maintaining normal plasma volume and osmotic balance. The purpose is the elimination of circulating toxic substances such as autoantibodies, immune complexes, proteins, and toxins. Albumin, the most abundant protein in blood plasma, is a multifunctional protein with roles in the (i) binding and transport of molecules (ii) extracellular antioxidant, (iii) immunomodulatory, (iv) anti-inflammatory, (v) anti-coagulant effects, and (vi) contributes to the maintenance of the normal capillary permeability [227]. Effect of ectopic albumin enhanced NPC proliferation by itself [228]. This work shifted the paradigm of blood heterochronicity away from the dominance of young blood factors and establishes that replacement of a large volume of old blood with a neutral age physiological fluid (saline supplemented with 5% purified albumin), is sufficient for most if not all observed positive effects.

In 2005, the first pilot study to validate this hypothesis was carried out in mild-to-moderate AD patients who underwent plasma removal with Albutein replacement (Albutein^®^, which does not contain detectable Aβ). In 2007, a Phase 2 trial (NCT00742417) [229] was conducted, and in 2012, a 14-month Phase 2b/3 trial, randomized, double-blind, placebo-controlled study (Alzheimer’s Management By Albumin Replacement, AMBAR, NCT01561053) evaluated the effects of different plasma replacement levels of albumin, with or without IVIGs [230]. When all treatment arms were combined the 66% lesser decline in the ADAS-Cog compared to placebo approached statistical significance (*p* = 0.06). On the ADCS-ADL, the treated group declined 52% less than those on placebo (*p* = 0.03). A low rate of adverse events was reported. In CSF, Aβ42 was stable in treated patients while decreasing in sham-treated patients and phosphorylated and total tau increased less in treated patients than in controls. Recently, inflammatory biomarker analysis in serum and CSF from the AMBAR trial participants have been presented in different conferences showing significant changes after TPE [231]. Indeed, PE with albumin replacement was associated with fewer deleterious changes in subcortical structures and less metabolic decline compared to the typical of the progression of AD [232]. Despite initial clinical results obtained with TPE in AD patients are encouraging, it should be noted that these studies, including the AMBAR trial, did not require biomarker-proven brain Aβ deposition at entry. In the AMBAR study, 28% of screened patients did not show evidence of Aβ brain deposition, meaning that these patients had an underlying reason for their dementia other than AD.

A 14-week, double-blind, placebo-controlled study (PLASMA, NCT02256306) evaluated the safety and tolerability of infusions of young fresh frozen plasma (250 mL) in mild-to-moderate AD patients [233]. Plasma infusion recipients demonstrated statistically significant improvements compared to placebo on functional measures but not on cognitive or clinical global measures. GRF6019 is a human plasma protein fraction depleted of coagulation factors and gamma globulins and containing about 400 proteins believed essential to the beneficial effects of whole plasma. GRF6019 is being evaluated in a clinical trial (NCT03520998) [234,235].

However, it remains to be discovered whether and which plasma factors would be active enough to influence neurogenesis or cognition at small doses when added to an aged circulation, and would be able to cross the blood–brain barrier to have positive or negative central effects.

## 8. Discussion

Developing effective treatments for conditions whose causes are still unclear is a difficult but necessary task [236]. The NIA–AA framework used for the diagnosis of AD is undoubtedly centered on amyloid β and tau, in which a deterministic chain of events leads from Aβ and then tau deposition to neurodegeneration and progressive cognitive impairment [237]. Subsequently, the current therapeutical approach for AD is mainly driven by the amyloid hypothesis. This model fits autosomal dominant AD (1% of total AD cases) but is less applicable to sporadic AD. A pathology study showed that about two-thirds of patients with dementia show co-morbid molecular pathology in addition to plaques and tangles, namely α-synuclein aggregates, insoluble aggregates of TAR DNA-binding protein 43, and vascular pathology together with chronic inflammation [238].

What are the lessons from the clinical trials conducted so far in AD? First, earlier initiation of the use of pharmacologic agents in early AD, or, if plausible, even before the onset of symptomatic changes may be necessary and desirable to increase the efficacy of anti-Aβ DMT. Anti-amyloid Phase 3 drug trials have moved to the early stages of AD (prodromal or preclinical stages) and thus we need to improve an early prediction (i.e., family history and the polygenic risk score of each subject), and detection (i.e., using fluids such as plasma and digital biomarkers). Second, a better understanding of the MoA and molecule optimization is required to lower the side effects of treatments (i.e., ARIAs, ADA). Third, the choice of therapeutic targets should be adjusted to the disease stage and patient subtype (i.e., endophenotypes). In an ideal world, patient characterization should be performed before inclusion in any clinical trial (i.e., genetic background, Aβ and p-Tau species, ApoE genotype, BACE and QC activity, cytokines profile, etc.) improve the options of success [239]. Fourth, AD as a multifactorial disease seems to require multi-targeted therapies, affecting several aspects of pathology (Aβ, Tau, glucose metabolism, inflammation, glycated proteins, vascular damage, etc.). If AD is the result of a cumulative effect of genetics and different comorbidities accumulated during 65 years, are we asking too much from a single intervention? The observation of moderate effect on cognition despite drastic amyloid brain reduction after anti-Aβ treatment suggests that more than 50% of the clinical progression is independent of Aβ deposition. Effects observed after the amyloid pathway together with positive results described in clinical trials targeting tau species and pro-inflammatory cytokines set the stage for alternative therapies and combined treatments [240]. Fifth, the delivery route of therapeutic agents has to be selected to ensure their efficient delivery to the right place and at the right time [241]. Sixth, novel hypotheses for AD are under investigation and should be taken under consideration (i.e., atuzaginstat, a gingipain inhibitor, has demonstrated promising results in AD patients with periodontitis, and GV-971, an algae extract that improves inflammation and reduces Aβ aggregation via alteration of intestinal bacteria).

Finally, several studies have analyzed the preparedness of healthcare systems to handle the potential caseload if a DMT for AD became available in 2021 [242]. The current AD drugs under regulatory agencies evaluation, would require an average waiting time of 19 months between seeking diagnosis and infusion delivery and require extremely high costs (i.e., specialist assessment, neuroimaging, CSF analysis) of the therapy for the healthcare system and weekly or monthly infusions. For this reason, it is mandatory to implement real-world strategies to facilitate the implementation of agile patient analysis and diagnosis (i.e., telematics assessment of the cognitive state, blood-based biomarker assays) and affordable and worldwide scalable treatments.
